# Single-Nucleotide Polymorphism Markers from De-Novo Assembly of the Pomegranate Transcriptome Reveal Germplasm Genetic Diversity

**DOI:** 10.1371/journal.pone.0088998

**Published:** 2014-02-18

**Authors:** Ron Ophir, Amir Sherman, Mor Rubinstein, Ravit Eshed, Michal Sharabi Schwager, Rotem Harel-Beja, Irit Bar-Ya'akov, Doron Holland

**Affiliations:** 1 Department of Fruit Tree Sciences, Agricultural Research Organization, Volcani Center, Bet Dagan, Israel; 2 Department of Fruit Tree Sciences, Agricultural Research Organization, Newe Ya'ar Center, Ramat Yishai, Israel; United States Department of Agriculture, United States of America

## Abstract

Pomegranate is a valuable crop that is grown commercially in many parts of the world. Wild species have been reported from India, Turkmenistan and Socotra. Pomegranate fruit has a variety of health-beneficial qualities. However, despite this crop's importance, only moderate effort has been invested in studying its biochemical or physiological properties or in establishing genomic and genetic infrastructures. In this study, we reconstructed a transcriptome from two phenotypically different accessions using 454-GS-FLX Titanium technology. These data were used to explore the functional annotation of 45,187 fully annotated contigs. We further compiled a genetic-variation resource of 7,155 simple-sequence repeats (SSRs) and 6,500 single-nucleotide polymorphisms (SNPs). A subset of 480 SNPs was sampled to investigate the genetic structure of the broad pomegranate germplasm collection at the Agricultural Research Organization (ARO), which includes accessions from different geographical areas worldwide. This subset of SNPs was found to be polymorphic, with 10.7% loci with minor allele frequencies of (MAF<0.05). These SNPs were successfully used to classify the ARO pomegranate collection into two major groups of accessions: one from India, China and Iran, composed of mainly unknown country origin and which was more of an admixture than the other major group, composed of accessions mainly from the Mediterranean basin, Central Asia and California. This study establishes a high-throughput transcriptome and genetic-marker infrastructure. Moreover, it sheds new light on the genetic interrelations between pomegranate species worldwide and more accurately defines their genetic nature.

## Introduction

Pomegranate (*Punica granatum* L.) belongs to the family Lythraceae and is grown in many regions worldwide. Wild pomegranates (*P. granatum* L. or *Punica protopunica*) have been reported as native to India [1], Turkmenistan [2], Iran [3] and Socotra [4]. The pomegranate fruit is considered to be highly valuable due to its health-beneficial effects, appealing taste and pleasing esthetics. Recent scientific publications have focused mainly on its health-beneficial characteristics and mechanisms of action in human and animal model disease systems. The health-promoting activity attributed to various parts of the pomegranate fruit, flower and bark included anticancer, antidiabetic, antiviral and antimicrobial functions [5]–[7]. In addition, consumption of pomegranate juice has been shown to reduce blood pressure and serum fatty acid concentration [8], [9]. Studies of the metabolites produced in pomegranate include mainly ellagitannins and gallotannins, ellagic acid derivatives, flavonoids, organic acids, fatty acids, triglycerides, sterols, terpenoids and alkaloids [10], [11].

In contrast to the vast knowledge about its health-beneficial activities, genetic studies of the pomegranate tree are based on insufficient information. The genetic diversity of pomegranate has been studied by several groups from Turkey [12], Iran [3], [13], [14], India [15], Tunisia [16], [17], Spain [18] and China [19], [20]. Those studies suggested classifying pomegranate accessions based on simple-sequence repeats (SSRs) [21], random amplification of polymorphic DNA [12], [22], [23], amplified fragment length polymorphisms [19], [24], 28S rDNA [18] or internal transcribed spacers [3]. Most of the analyses were performed on local accessions except for one report which included a limited number of accessions introduced from different countries [21]. Two studies [15], [16] that used broader collections showed that plant material from different regions is genetically distinguishable. Other studies have compared wild, semi-wild and domesticated pomegranate accessions [3], [15]. Overall, these studies identified substantial polymorphism among pomegranate accessions and were successful in splitting the local collections into several closely related subgroups. However, this was not based on a broad genomic infrastructure. Progress in the genomic area has been shown by a recent study that produced partial transcriptome data from pomegranate fruit peel mRNA using deep sequencing with the Illumina platform [25].

The currently available genomic resources for pomegranate cannot support modern breeding efforts such as marker-assisted selection or elaborate molecular studies. Next-generation sequencing (NGS) methods provide powerful tools for non-model organisms. In particular, 454-GS-FLX Titanium technology provides long reads along with considerable yield [26]. Transcriptome sequencing is one solution for large (>0.5 Gb) genomes that enables reducing complexity by focusing on genic regions. Sequencing the transcriptome using NGS technologies rapidly generates large sets of genetic markers linked to genes. Therefore, many studies have used this technology to investigate the behavior of wide gene-expression profiles in non-model organisms such as *Ammopiptanthus mongolicus*, bitter melon fruit and root, peony, watermelon and carrot [27]–[32]. Moreover, the overwhelming information provides an opportunity to characterize a crop's transcriptome in terms of its genetic variation and repertoire of gene functionalities [33]–[36]. The most useful markers are SSRs and single-nucleotide polymorphisms (SNPs). These genetic markers are abundant and distributed throughout the genome. On average, there is 1 SNP per 200–500 bp [37], [38] and 1 SSR per 1.5–5 Kbp [39]. Therefore, the integration of SSR and SNP discovery in cDNA sequencing using NGS results in an overwhelming number of useful markers. In the previously published transcriptome from pomegranate, only 115 SSR markers were identified but no SNP markers[25].

In this work, we used high-throughput sequencing to discover major parts of the pomegranate transcriptome. We used this genomic information to compile a genetic-variation resource. A sample of the discovered genetic variation was utilized to learn about the genetic basis of the broad pomegranate germplasm at the Agricultural Research Organization (ARO) [40].

## Materials and Methods

### Plant material

The ARO's pomegranate germplasm collection is located at the Newe Ya'ar Research Center in northern Israel (http://igb.agri.gov.il/main/index.pl?page=22). In the present study we analyzed 105 pomegranate accessions (Table S1) originating from different geographical locations all over the world and in particular, Israel [40]. Two *P. granatum* accessions with differing phenotypes were chosen to reconstruct the pomegranate transcriptome: ‘Nana’ (P.G.233–244) is a *P. granatum* var. Nana seedling characterized as a dwarf, conditional dormant pomegranate. ‘Nana’ has a very small and sour fruit with hard seeds and a green to red peel. Because ‘Nana’ is so distinct, it was recognized in one study as a third species of the genus *Punica* (*P. nana* L.) [41]. ‘Black’ (P.G.127–28), is a domesticated cultivar characterized as a deciduous normal-sized tree with a very distinct deep-purple peel. The ‘Black’ accession has a fruit of medium size, with sweet taste and soft seeds.

### Accession phenotyping

Phenotypic classification was based on a phenotypic description of the ARO collection at the Newe Ya'ar Research Center. The collection was established in 1978 and new accessions have been added to the orchard ever since. Once each of the accessions reached the fruiting stage, the tree and fruit were characterized for at least 5 years. Characterization included, among other traits, date of maturity (when fruit reach edible quality), fruit size (weight and diameter), peel and aril colors (visual description) and taste (organoleptic description). Five mature fruits were harvested from two different trees of each accession, from different parts of each tree. Mature fruits were chosen by their combination of aril and peel color and by tasting for astringency and sourness. Fruit size, weight and diameter of the fruits were averaged. Colors and taste were described by observing and tasting the fruit.

### RNA extraction for 454-GS-FLX sequencing

Total RNA was extracted from 3 g of ground tissue according to Meisel et al. [42], followed by two additional sodium acetate/ethanol precipitations. RNA was extracted from leaves, roots, flowers (petals, anthers, ovaries) and fruit at developmental (Stage 3; [43]). For each accession, equal amounts of total RNA from each tissue were mixed.

### DNA extraction

The DNA-extraction protocol was based on Porebski et al. [44] with a few modifications. Young leaves from 105 pomegranate accessions were used for DNA preparation, 0.5 g resuspended in 6 ml extraction buffer. The chloroform–octanol solution was replaced with chloroform–isoamyl alcohol. DNA was precipitated with sodium acetate instead of sodium chloride.

### De novo transcriptome assembly and functional annotation

Raw files of sequence reads were preprocessed by “SFF_extract” (http://bioinf.comav.upv.es/sff_extract/) and arguments for removing the adaptors and clipping the poly-A were applied. Reads from 454-FLX GS Titanium were assembled by a stable version of MIRA, v3.2 [45]. For the MIRA run, we used the “denovo,est,normal,454” set of “'Do-What-I-Mean” (DWIM) parameters. The complete set of sequence reads of ‘Black’ and ‘Nana’ were uploaded to SRA (SRS516503 and SRS516504, respectively.

All contigs were searched for open reading frames (ORFs) by the “getorf” program from the EMBOSS package[46]. The longest ORF with start and stop codons was chosen for each contig with a minimum cutoff of 50 amino acids. ORFs were run against *Eucalyptus grandis* proteins downloaded from Phytozome v9 [47]. Estimates of the homologous-segment fraction between pomegranate ORFs and eucalyptus proteins were calculated by cover index (CI) as CI  = 2*HSP/(QL+HL) where HSP is the high-scoring segment pair (i.e., the blast alignment), QL is the query length (i.e., pomegranate ORF) and HL is the hit length (i.e., the eucalyptus protein).

A sequence-similarity search of contigs was run against the nonredundant protein (nr) database using blastx with a filter of e-value <10^−5^. Best hits were further mapped to GO-slim by Blast2GO [48] and only hits with Blast2GO annotation score >55 were scored. Mapping the pomegranate fruit peel transcripts to the transcripts of the pooled tissues in this study was performed by blast search for all transcripts of peel against pool and vice versa, and selecting the reciprocal best hits.

### SNP and SSR discovery

The SNP position and the coverage of each nucleotide allele were derived from the MIRA contigs output file. The coverage was only counted for nucleotides with PHRED-scale quality >30 [49]. Only positions whose nucleotide allele had a coverage of ≥3 were considered valid SNPs.

SSR scanning was performed on the 67,532contigs. MIcroSAtellite (MISA) identification tools and SciRoKo were run with default parameters.

### SNP subset for genetic analysis

A subset of 480 SNPs included SNPs with the highest coverage restricted to a single SNP per contig. SNP assays for all 480 SNPs were developed by Fluidigm Corporation (http://http://www.fluidigm.com/snp-genotyping.html) based on variation data between cultivars ‘Nana’ and ‘Black’. The SNP assays were used to screen the 105 accessions' DNA samples by running on FR48.48 arrays of the EP1 Fluidigm platform according to the manufacturer's instructions (http://www.fluidigm.com). To exclude bad samples and failed marker assays, samples that had more than 10% “No Call” and assays with more than 30% “No Call” were removed. The remaining subset was submitted for the downstream analysis.

The polymorphism information content was calculated as [50]

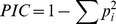
(1)where i is the i^th^ allele.

### Germplasm accession classification and diversity

To assess the relationship between pomegranate accessions, we estimated the genetic distance as D =  1-proportion of shared alleles (PSA). PSA was calculated as
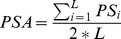
(2)where PS is the proportion of shared alleles for each locus and L is the total number of loci [51].

Hierarchical clustering was performed on a pairwise D distance matrix and the “ward” agglomerative method [52] was applied. The confidence limits of the tree topology were calculated by applying bootstrap method (1,000 resampling of loci). To count the number of bipartitions fit to the tree we used the “ape” R-package [53], [54] and presented the bootstrap values as percentages.

The subpopulation structure underlying the germplasm collection was estimated by running a simulation of STRUCTURE software v2.3.3 [55] with 5,000 burn-in periods and 50,000 repetitions. The number of populations, K, was inferred by running the simulation of K = 1 to K = 10 (20 runs for each K) and using the likelihood method of ΔK [56].

The fixation indices F_S_ and F_ST_ [57] were calculated as
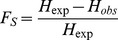
(3)where F_S_ is the fixation index of each subpopulation, H_obs_ is the observed heterozygous types and H_exp_ is the estimated heteozygosity under Hardy-Weinberg equilibrium (HWE),
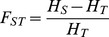
(4)where F_ST_ is the genetic differentiation of a subpopulation due to genetic drift, H_S_ is the weighted average of all subpopulations' expected heterozygozity, and H_T_ is the expected heterozygosity in the entire population (germplasm collection).

## Results

### Pomegranate transcriptome reconstruction

**Figure 1:**
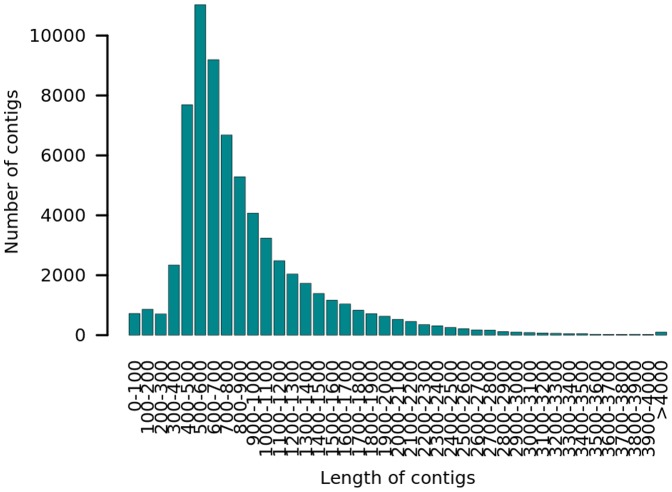
Distribution of contig lengths from both accession assemblies. Reads of cDNA sequencing by 454-GS-FLX Titanium technology were assembled by MIRA assembly program. The distribution of consensus contig lengths is drawn as 100-bp long bins.

We extracted mRNA from two *P. granatum* L. cultivars: ‘Nana’ (P.G.233–244) and ‘Black’ (P.G.127–28), from the ARO pomegranate collection. These accessions are very different phenotypically and are therefore assumed to be genetically different as well. ‘Nana’ is characterized as a dwarf pomegranate that has a temperature-conditional dormancy period. ‘Black’ is an edible deciduous cultivar with purple (almost black) peel color. To avoid over-representation of tissue-specific gene expression, mRNA samples of leaves, roots, flower parts (petals and reproductive organs) and fruits at developmental stage 3 [43] were pooled together. For each cultivar accession, cDNA pooled from these tissues was sequenced by the 454-GS-FLX Titanium platform, a pool per accession per half plate. The sequence results yielded a total of 755,519 and 728,665 reads for ‘Nana’ and ‘Black’, respectively, in which most of the reads (80.08–82.62%) from both samples were successfully assembled (Table 1). Half of the contigs were longer than 707 and 719 bp, respectively. The joint assembly of reads of both accessions yielded a median 714 bp, suggesting no preference for separate assemblies. The skewness to the right (positive skewness values) of contig-length distributions indicated that these distributions are asymmetric, i.e., there is a tail of long contigs (Figure 1). The skewness values of the joint assembly, the ‘Nana’ accession assembly and the ‘Black’ accession assembly were 2.16, 1.16 and 1.18, respectively, indicating that the joint assembly generated longer contigs. We therefore focused on the joint assembly as a reference for further analysis.

**Table 1:** Assembly parameters for ‘Nana’ and ‘Black’ accession reads.

Assembly	Number of reads	Reads filtered out (%)	Number of contigs	Contig length (N50)	Contig length (average)	%GC (N50)
Black	728,665	19.15	43,027	719	862.5	47.32
Nana	755,519	17.38	46,734	707	849.5	47.33
Both	1,484,183	18.25	67,532	714	880.2	47.36

### Functional annotation of pomegranate transcriptome

**Figure 2:**
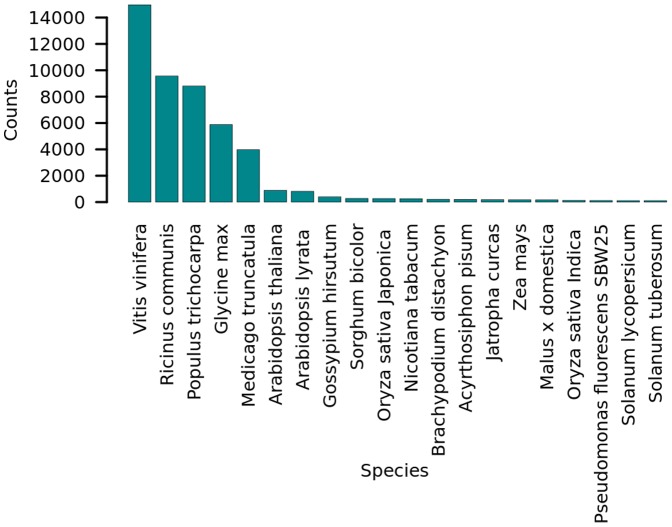
Distribution of top homologs according to their taxonomy. The assembled contigs were submitted to sequence-similarity search (blastx) against the nonredundant protein (nr) GenBank database. The frequency of best-hit species was counted and is illustrated in a bar plot. Only species with more than 100 hits are presented.

To explore the gene repertoire in the pomegranate transcriptome, a DNA–protein similarity search (blastx) against the nr database was performed. Mapping blast hits to gene ontology (GO) and downstream annotation analysis were performed using Blast2GO [48], [58]. Out of 67,532 contigs, 58,473 (86%) included an ORF, 54,838 (81%) had a significant hit (e-value <10^−5^) and 45,187 (67%) passed the minimum blast2 GO-annotation score of 55, which means that they were mapped to one of the GO categories (Table S2). Most of the homologous protein hits in GenBank were plants (99%) (Figure 2), with 81.27% of the hits being proteins of *Vitis vinifera*, *Ricinus communis*, *Populus trichocarpa*, and *Glycine max*. This suggests that most of the functional annotation derived from the plant-homologous hits, and that the DNA sample was not contaminated.

Annotation relies on sequence similarity of the mRNA products to homologous proteins with functional descriptions. The joint assembly produced long, but not essentially more informative contigs. Therefore, we estimated whether the proteins derived from the ORFs of the pomegranate assembly include most of the coding sequence. A blast search was run against 46,315 proteins of *Eucalyptus grandis,* a sister taxon in the Myrtales clade, and the ratio of blast alignment to length of the pomegranate query and eucalyptus hit was calculated (see Materials and Methods). The ratio was notated as CI. Half of the contigs included ORFs with CI ≥0.86, and 90% of the contigs included ORFs with CI ≥0.41. In comparison, in the pomegranate transcriptome from the peel [25], half of the contigs included ORFs with CI ≥0.38 and only 10% of the contigs included ORFs with CI ≥0.85.

**Figure 3:**
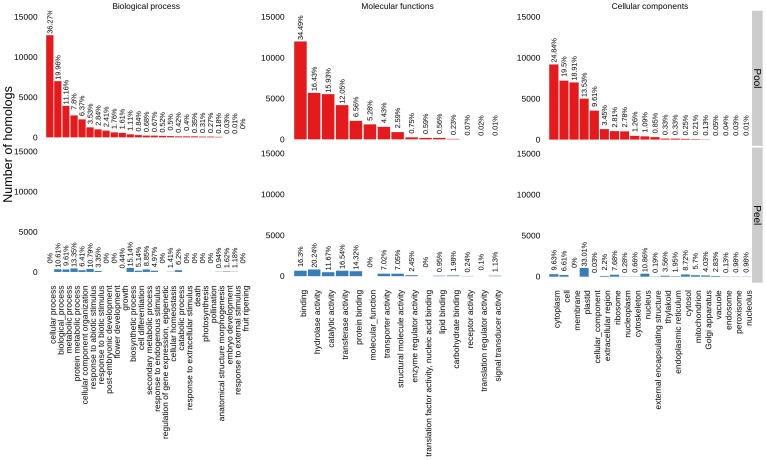
Pomegranate gene ontology categories. Contigs were annotated by blast hits against the nr database and mapped to GO categories by Blast2GO. The distribution of contigs into the GO categories biological processes, molecular functions, and cellular components is plotted for a pool of tissues (root, leaf, flower and fruit developmental stage 3; red bars) and from peel (turquoise bars).

The cDNA sequencing was performed with multi-tissue samples. Therefore, the knowledge that could be derived on gene functionality was essentially non-tissue-specific. However, it would be interesting to investigate whether pomegranate has a bias toward specific functions. The pomegranate contigs mapped to 323,654 GO categories. Where the contigs were mapped to more than one category, the most specific category was chosen to convert the “one to many” to “one to one” mapping. Many studies use the GO-Slim set of GO categories [28], [29], [59]–[61]. Plant GO-Slim represents a set of GO categories which is most relevant to all plants. Contigs mapped to the root of the ontologies can be considered unknown. Notably, the root category was not the most frequent on any of the ontology graphs (biological processes, molecular functions, and cellular components) (Figure 3), indicating successful annotation. The most frequent (30%) informative biological processes were various types of metabolic processes, cellular organization, and responses to abiotic and biotic stimuli. The most frequent molecular functions were various binding functions (41.05%), and hydrolase (16.43%), catalytic (15.93%) and transferase (12.05%) activities.

A comparison between the transcripts from the pooled pomegranate tissues and the transcripts from the pomegranate peel [25] enabled us to outline the differential scheme of biological processes and molecular functionalities between peel and other tissues (root, leaf, flower and fruit at a developmental stages). The intersection of the transcripts from the pool with those from the peel was mapped by reciprocal blast hit of the contig sequences, resulting in 5,943 corresponding transcripts. Similar to the annotation of transcripts from the pool of several tissues, full ORFs derived from the peel transcripts were submitted to the Blast2GO pipeline against “nr” (Figure 3). Peel seemed to lack transcripts corresponding to flower development, embryo development, pollination, photosynthesis, death, response to extracellular stimulus, and epigenetics. This illustrates the advantage of a pool in which flower tissues and fruit development stages contribute genes that are not abundant or are absent in the peel. Furthermore, plastid genes dominated the peel tissue, whereas cytoplasmic and membranal genes were contributed by the pooled tissues.

### SSR discovery

**Figure 4:**
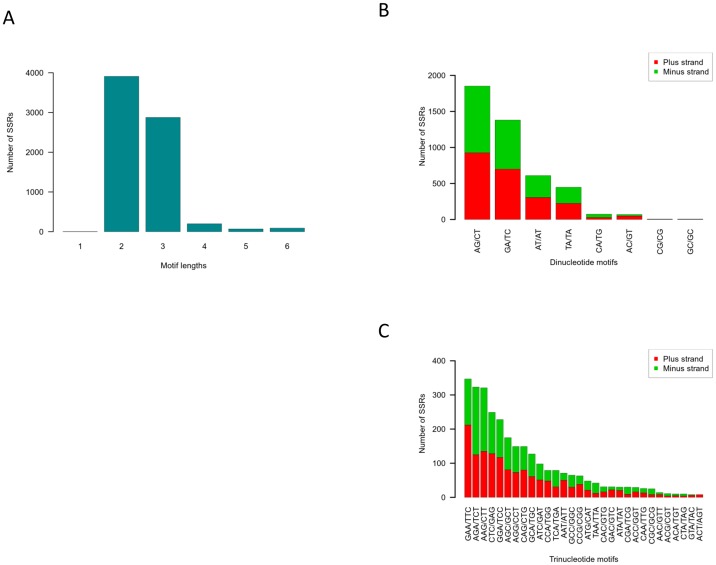
SSR length and motif distribution. The number of mono- to hexanucleotide SSR motifs was counted (A). The nucleotide compositions of the most frequent motifs (di- and trinucleotide motifs) was determined for each type and is illustrated in a bar plot for dinucleotide (B) and trinucleotide (C) motifs. Motifs that are reverse-complementary were plotted one above the other as “plus strand” (red) and “minus strand” (green).

Although SNPs are more abundant than SSRs and more economic per marker, the latter are still valuable for genetic studies when focusing on a small number of markers [62]. We therefore screened for SSRs in all contigs by running two different SSR-detection programs: MISA [63] and SciRoKo [64]. Whereas the former is faster and more popular, the latter is expected to be more robust for SNPs in the SSRs. The number of SSRs found in the entire set of contigs (67,532) was 10,330 and 12,309 for MISA and SciRoKo, respectively. The robustness of SciRoKo is reflected in a higher proportion of SSRs with four and five motifs. However, we assumed that the intersection of the two approaches for SSR identification would decrease the number of false SSRs. We therefore crossed the lists of SSRs based on the SSR motif and its start position in a contig. The intersection list included 7,155 putative SSRs (Table S3). The SSR motifs consisted of mono- to hexanucleotides. However, most were di- and trinucleotide motifs, totaling 3,910 (54%) and 2,876 (40%), respectively, whereas tetranucleotide motifs were an order of magnitude less abundant (201; 3%) (Figure 4A). The preponderance of (AG/TC)n and (GA/CT)n for dinucleotide motifs, and of (GAA/TTC)n, (AGA/TCT)n and (AGG/CCT)n for trinucleotide motifs is altogether unique to pomegranate (Figure 4B and 4C) and is consistent with a previous study in which 117 pomegranate SSR markers were reported in AG/TC-rich regions [21]. Moreover, the (AG/TC)n motif was found to be the most frequent among 115 SSRs in the pomegranate fruit-peel transcriptome [25]. Nevertheless, the (AAG/CTT)n motif is most abundant in many closely related species such as *Prunus* spp. [65], *Eucalyptus grandis* [66] and Cucurbitaceae species [59], as well as Brassicaceae speceis [67]. In summary, the uniqueness of pomegranate lies in the order of trinucleotide-motif frequency: (GAA/TTC)n was most abundant, (AGA/TCT)n was the second and (AGG/CCT)n was the third most abundant trinucleotide motif. In melon, for example, (AAG/CTT)n is the most abundant motif whereas in *Prunus* species, (AAC/GTT)n is the most frequent. In *E. grandis*, (AAG/CTT)n is the second most abundant while the (GGC/CCG)n motif is the most frequent.

### SNP discovery

**Figure 5:**
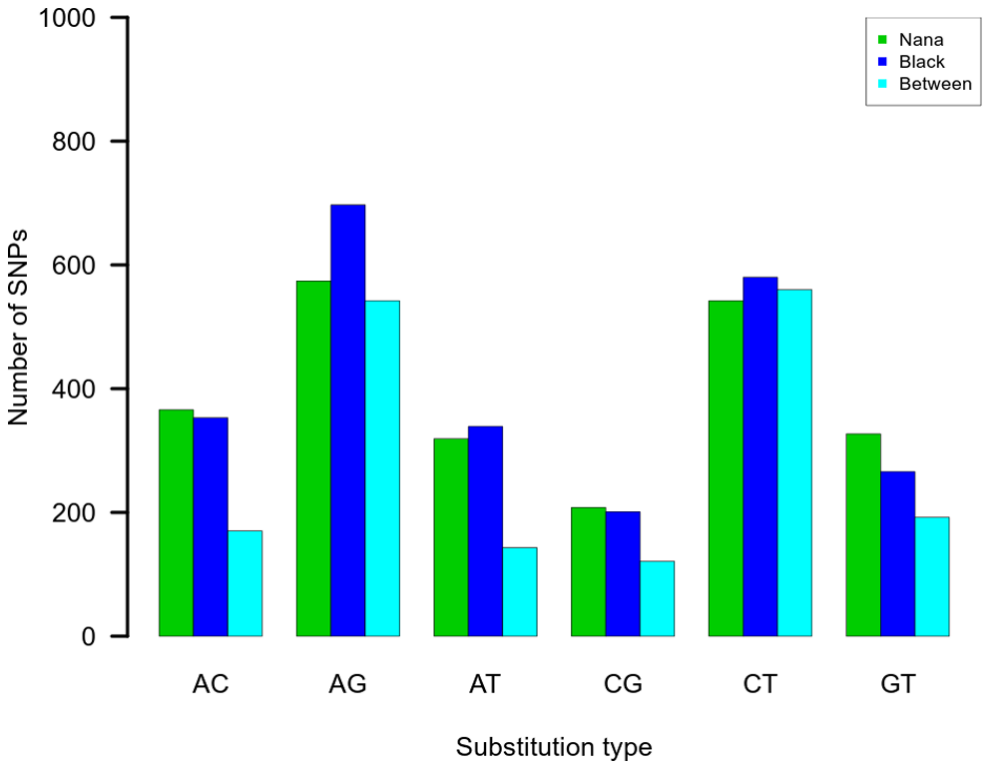
Distribution of SNP substitutions within and between ‘Nana’ and ‘Black’ accessions. The frequency of nucleotide substitutions was counted in ‘Nana’ (green) and ‘Black’ (blue). Where ‘Nana’ was homozygous for one allele and ‘Black’ was homozygous for another, it was counted as a between-accession SNP (turquoise).

SNP discovery has become routine in high-throughput parallel sequencing [33], [34], [68], [69]. We investigated the polymorphism of SNPs between the two accessions where each accession was homozygous for a different allele, and within each accession. To avoid sequencing errors, we relied on minimal resequencing coverage at the SNP locus. Therefore, the coverage of each allele was only counted for good-quality base calls and we filtered out base-call variants if one of the alleles was covered (resequenced), using 3X coverage as the cutoff. The number of SNPs for ‘Nana’ and ‘Black’ was 2,336 and 2,436, respectively, whereas the number of SNPs between the two accessions was 1,728. For all three sets of SNPs, transition substitutions were more frequent than transversion substitutions. The highest transition-to-transversion (t/v) ratio was estimated between the accessions (3.5), whereas the t/v ratios within ‘Nana’ and within ‘Black’ were1.8 and 2.2, respectively. Transitions are expected to be more frequent than transversions [70], [71]. However, this t/v ratio was higher for SNPs between accessions rather than within accessions. The increase in the ratio was due to decreasing transversion substitutions of these SNPs, rather than increased transitions (Figure 5). This underlies the fact that the SNPs were sampled mostly from coding sequences and the selection on transversion substitutions in a coding region is higher than on transition substitutions. Moreover, this effect increased when comparing the t/v ratio between the two cultivars.

### Germplasm diversity

Our major interest was to understand the genetic structure of the ARO's pomegranate germplasm collection at Newe Ya'ar Research Center (Table S3;[40]). Thus, 480 SNPs were selected from the transcriptomes of the ‘Nana’ and ‘Black’ accessions and used to survey the genotypes in the ARO germplasm collection by Fluidigm-EP platform. To ensure even genome distribution of high-confidence markers, SNPs with the highest read coverage in each contig were selected. Because our criteria were not biased toward a specific type of SNP, we expected no change in the t/v ratio. Indeed, the proportion of transition SNPs was 0.6. First, the marker diversity was estimated by calculating the polymorphism information content (PIC) for each SNP. Half of the SNPs were highly diverse (PIC ≥0.43), indicating that the selected SNPs are highly diverse in the ARO pomegranate germplasm. Only 10.7% of the SNPs showed minor allele frequencies (MAF <0.05), consistent with previous studies [19], [21], suggesting that the pomegranate genome is highly diverse. MAF SNPs are an uninformative subset and were therefore filtered out, as were SNPs whose genotype called as heterozygous for all germplasm accessions. Consequently, a set of 346 SNPs was retained for further analysis.

### Relationship among germplasm accessions

**Figure 6:**
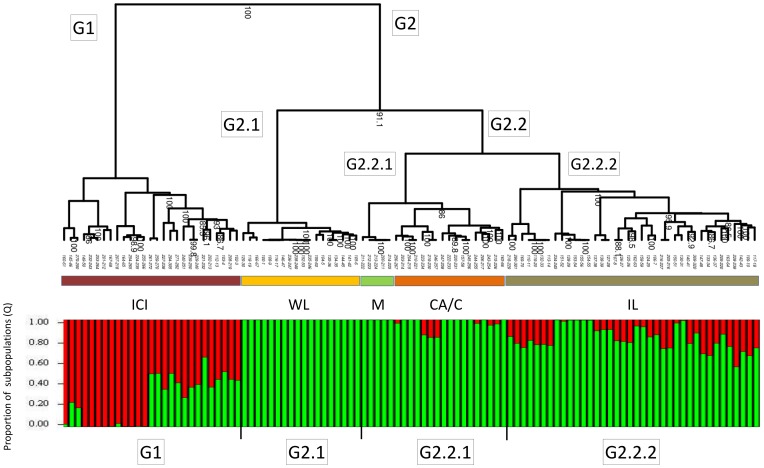
Dendrogram and genetic structure of 105 accessions in the ARO pomegranate germplasm collection. Genotyping of 105 pomegranate accessions from the ARO collection was performed with 346 SNPs. The genotyping results were used to classify the accessions into subpopulations and reveal their genetic structure. Classification was performed by drawing a dendrogram based on 1-PSA as genetic distance. Only confident branches with bootstrap values above 85 were assigned. The two major groups are notated as G1 and G2, and G2 was further divided into G2.1 and G2.2, giving altogether three groups of related accessions. The colored horizontal bars correspond to phenotypically homogeneous pomegranate groups of accessions: India-China-Iran (ICI), “Wonderful-like” (WL), Mollar (M) Central Asia and California (CAC), and Israel (IL). Genetic structure was revealed by STRUCTURE program with K = 2 as found by simulation and δK likelihood method. The two subpopulations (K = 2) are represented as green and red bars. The division of STRUCTURE's Q-value bar plot into four (vertical lines) corresponds to the four major significant clusters in the dendrogram.

The ARO germplasm collection includes local accessions and several accessions introduced from foreign countries, including India, China, Central Asia, USA, Spain, Turkey and the Mediterranean region (Table S1; [40]). However, the genetic relationships among the 105 examined accessions and their genuine geographical origins are still uncertain. Thus, classifying the accessions only by their origin might be misleading. To classify the accessions into groups of kinship based on their genetics, we calculated the pairwise genetic distances as 1- PSA [51], [72] among all accessions, and a hierarchical classification tree was reconstructed (Figure 6). Confidence in the tree topology was obtained by bootstrap method (1,000 resampling). As a complement, the STRUCTURE program was used to reveal the number of subpopulations composing the genetic structure of the ARO germplasm collection. Therefore, we applied the δK likelihood method [56]. The maximum ΔK corresponded to K = 2, suggesting that there are two major subpopulations [56]. The proportion of subpopulations (Q) is presented in Figure 6 in comparison to the dendrogram, which classifies the collection into groups of closely related genetic accessions (hereafter called groups and subgroups).

**Figure 7:**
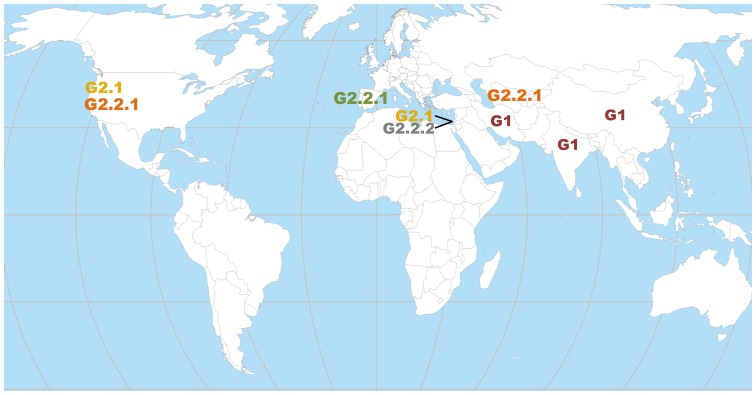
Worldwide distribution of the pomegranate genetic groups. The genetic groups as clustered together by the genetic dendrogram were located on a world map by geographical regions. Genetic groups and subgroups are colored as in Figure 6.

The classification tree (i.e., dendrogram) revealed two major groups of accessions (G1 and G2); G2 was further divided into two subgroups (G2.1 and G2.2). Strictly speaking, the dendrogram implied altogether three statistically significant groups (bootstrap value >90%). Group G1 (P.G.160–61 to P.G.102–3) includes ornamental, inedible pomegranate accessions such as *P. granatum* var. Nana seedlings (e.g., P.G.149–50 and P.G.167–68), as well as accessions from India, China and Iran (ICI; e.g., P.G.145–46, P.G.202–213, P.G.102–3; brown bar, Figure 6). The G2 group (P.G.129–30 to P.G.117–18) includes accessions from the Mediterranean region, Central Asia and California. This group includes domesticated pomegranate cultivars and is further divided into two main subgroups: subgroup G2.1 (P.G.129–30 to P.G.105–6; yellow bar, Figure 6) consists mostly of the “Wonderful-like” (WL) accessions and seems to be genetically and phenotypically uniform. Most of the WL accessions were collected in Israel and contain divergent landraces and mutants [40]; subgroup G2.2 (P.G.211–222 to P.G.214–225) includes accessions originated from the Mediterranean basin, Central Asia and California and was split into two further subgroups: G2.2.1 (P.G.221–222 to P.G.165–66) and G2.2.2 (P.G.218–229 to P.G.117–18), although this separation was statistically insignificant. Nevertheless, G2.2.1 includes accessions introduced from Spain [“Mollar” (M); green bar, Figure 6] and accessions introduced from Central Asia and California (CAC; orange bar, Figure 6), both of which are more homogeneous than the rest of the G.2 members. In contrast, G2.2.2 includes slightly more of an admixture of accessions. In this subgroup, the exception is a few accessions that are genetically homogeneous and have similar phenotypic characteristics. For example, the “Early Red Sweet” (ERS) accessions (P.G.234–245 to P.G.154–55), which are characterized by dark red color of the arils and peel, sweet taste and soft seeds, clustered together with high statistical confidence. A world view of the geographical location representing the suggested origin of each of the subgroups described in the dendrogram of germplasm accessions is presented in Figure 7. The groups' location on the world map illustrates that the two major groups in the dendrogram are separated by their origin.

### Genetic group diversity

The genetic flow within the germplasm collection can be retrieved by estimating the medians of the fixation index of a subpopulation (F_S_) for all SNP loci. We estimated the median F_S_ of each of the three subgroups defined based on the dendrogram (Figure 6). The F_S_ values deviated from the HWE with positive values of 0.41, 0.33, and 0.32 for G1, G2.1, and G2.2, respectively. These results indicate that the subgroups are much less heterozygous than expected under the HWE, probably due to a long period of cultivation. The median F_S_ value for G1 was surprising as it is expected to be composed of more ancient accessions. One might assume that they would be more diverse because they had more time to evolve. Our results seem to suggest that they were evolved from different founders that were not intercrossed. Moreover, subgroup G2.2.1 includes three different geographical origins. The calculation of F_S_ for the Spanish M accession and for CAC-originated accessions revealed that G2.2.1 is a mix of two separate subgroups. The median F_S_ of the M accession was 0.52 and that of the CAC accessions was 0.19. Next, the estimation of genetic differentiation among the three subgroups was calculated as F_ST_. The loci's F_ST_ values deviated only slightly from the HWE. Most of the loci (90%) deviated less than 5% and the median was only 1.2%. This suggests that the subgroup differentiation occurred recently.

## Discussion

The primary objective of this work was to generate a comprehensive genomic infrastructure of pomegranate (*P. granatum* L.). This was done by reconstructing the transcriptome from several tissues that originated from two genetically divergent pomegranate accessions. Once a solid genomic infrastructure was established, a sample of the genetic variation was selected to explore the genetic structure of our pomegranate germplasm collection.

### Transciptome assembly quality

We used 454-GS-Titanium as a strategy for the de novo assembly of a transcriptome from a non-model organism. This approach has been commonly used in the last few years [31]–[33], [35]. In the present study, this strategy was used to create a resource for SNP mining [33], in order to reveal the genetic diversity of the ARO pomegranate collection. The use of two putatively genetically different accessions was presumed to enable discovering the genetic variations reflected as SNPs and SSRs. For this purpose, mRNA from a variety of tissues from each of the accessions was extracted and pooled, as has been done previously in melon and eucalyptus [34], [69]. Aside from the fact that the transcriptome assembly in this study resulted in a high frequency of long contigs, and most (70%) of the contigs included ORFs resembling eucalyptus homologs (CI ≥0.7), 45,187 (67%) contigs were fully annotated by Blast2GO [48]. This suggests the establishment of a high-quality genomic infrastructure. The annotation process was performed against the nr GenBank protein database which contains a broad set of proteins from a variety of organisms. As expected, most homologous hits were plants, mainly rosids. However, the most frequent hits were from *Vitis vinifera* rather than the more closely phylogenetically related species *Eucalyptus grandis* and *Populus trichocarpa* [73]. A possible explanation for this is that the proportion of *V. vinifera* sequences in the nr database is higher than those of *Eucalyptus* and *Populus*.

### Pomegranate transcriptome

Pomegranate is a fruit tree that belongs to the family Lythraceae and is part of the order Myrtales. A comprehensive genomic study was recently performed on its relative tree, *Eucalyptus*, which is part of the Myrtales, and peach (*Prunus persica*) and pear (*Pyrus pyrifolia*), which are part of the family Rosaceae. It is difficult to compare functionality among transcriptome studies for three reasons: 1) the source of the mRNA is not uniform and tissue-specific expression can bias the comparison; 2) the annotation method can affect the functionality results (e.g. use of different GO levels); 3) corresponding results may be due to lack of specific information. For example, “metabolic processes” is one of the most frequent GO categories, regardless of species or type of GO annotation method [59], [74]–[76]. Therefore, we could not conclude on the uniqueness of a functionality related to pomegranate. However, in pomegranate, like in pear (∼20%) [75], reproductive processes and pollination are enriched as compared to other trees such as peach (∼2%) [76], eucalyptus (0%) [69] and olive (6%) [74]. We did, however, compare the transcriptome from a pool of tissues in this study and from peel in a previous study [25], and showed that the strategy of pooling different tissues can enrich the repertoire of genes.

SSR markers from transcribed regions are commonly used in crops for genetic analysis [77]–[79] and for cultivar classification of crops [80]–[82]. In this work, the resource of such markers for pomegranate was immensely broadened. The combination of frequent SSR motifs is known to be species-specific [83]. However, as expected, some of the frequent SSR motifs are common to closely phylogenetically related species. In pomegranate, for example, the most frequent dinucleotide motif is (AG/TC)n, which is common to *Prunus* and *Eucalyptus*, whereas the high frequency of (GAA/TTC)n is unique to pomegranate and is barely found in these other species. The resemblance of most SSR motifs to closely related species supports the validity of the long list of SSR markers obtained in this study.

SNP markers are more abundant within a genome and are cost-effective for genotyping. However, the discovery of SNPs is primarily dependent on the sequencing coverage [84]. This coverage is not evenly distributed when sequencing cDNA [85] and therefore, when using the allele coverage as a cutoff for selecting true SNPs, the number of SNPs drops to less than the number of SSRs. Thus a SNP-discovery study of a non-model organism that lacks either a genome or transcriptome reference should integrate de novo assembly followed by resequencing of deep sequencing [86]. One of the pitfalls of SNP discovery from de novo assembly is false identification of SNPs that are paralogous, i.e., that result from misassembly of reads from repetitive sequences in the genome. This might cause an overestimation of SNP frequency. Another pitfall might stem from the detection of SNPs in cDNA. Allelic-specific expression might cause extensive coverage by one allele but not by the other. Thus, setting high allele coverage may cause an underestimation of SNP frequency. In pomegranate, the t/v ratio is approximately 2 as in peach coding sequences [76]. The decrease in the transversion SNPs between the accessions is typical to genic regions [70], [71]. Transversions cause nonsynonymous substitutions which are subjected to deleterious selection in the coding sequence. This supports the assumption that ‘Nana’ and ‘Black’ accessions are genetically different and were separately subjected to recurrent selection.

### Germplasm accession kinship and diversity

Understanding the genetic structure of the pomegranate germplasm is crucial for studying the inheritance and breeding relevance of important agricultural traits. It is essential to understand the pomegranate collection's structure to avoid stratification and admixture when performing genome-wide association studies [87], as well as to avoid redundancy when crossing accessions. After filtering out the bad-call and noninformative SNPs, 71% informative markers were left. The dendrogram which was established in this study is based on the analysis of those 346 SNPs and surveys 105 accessions from the ARO pomegranate germplasm collection. This collection compiles many accessions originating from Israel and from several other countries, such as the USA, India, China, Iran and Turkey.

The genetic classification divides the germplasm collection into two statistically significantly distinct genetic groups: G1 and G2. The G1 group is composed of *P. granatum* var. Nana seedlings and its descendant accessions, in addition to accessions of Indian, Chinese and Iranian origin. It includes the Indian cultivar Bhagwa and all of the conditional dormant (evergreen) accessions. This group is defined as the ICI group (red bar, Figure 6). The division into G1 and G2 groups separates accessions originating in India from those originating in Israel, California, Spain and Turkey, as has been reported previously [21]. However, in this study, G1 includes additional accessions from Iran and China. The dendogram separates the pomegranate accessions into two general geographical regions (Figure 7). One branch spreads from the suggested origin of the pomegranate species toward the Far East, while the other spreads toward the West. One important aspect is the classification of wild pomegranate. A clear definition of the term “wild” in *P. granatum* species is missing. Some studies refer to ‘Daru’ pomegranates that grow in the forests on Himalayan slopes as wild pomegranates (e.g., [88]). These pomegranates are characterized by thorny bushes with very low quality sour fruits which resemble the fruits of ‘Nana’ [89].

In general, the G1 group is more admixed than the G2 group, which includes the rest of the accessions. The division that is close to the root of the dendrogram (i.e., the two major groups G1 and G2) corresponds to the subpopulations revealed by the STRUCTURE program analysis and global geographical origins, whereas the clusters that are closer to the leaves correspond the phenotypic and local geographical origins. The G2 group can be subdivided into two subgroups (G2.1 and G2.2), and further divided into four subgroups (WT, M, CAC and IL). Some of these groups are still highly distinct, both phenotypically and genotypically. The WL (G.2.1) group is characterized by large fruit with a sour–sweet taste and red peel and arils. Some accessions do not correspond to this description, but those are most probably mutations of WL types as they have been reported to be sports of ‘Wonderful’ (e.g. sweet P.G.105–6). ‘Wonderful’ is present in a large number of landraces in the Mediterranean region. Moreover, there are reports of phenotypes similar to that of ‘Wonderful’ that have been described in the Mediterranean region for many years, such as ‘Red Lufani’ [90]. This suggests that the Mediterranean region is the geographical origin of this group. The genetic structure of the WL group is homogeneous (green in STRUCTURE analysis), in contrast to most other accessions in the second subtree of the G2 group. This suggests that the WL group underwent a process of stratification. Some of the subclusters of G2.2 branch have a prominent geographical profile or phenotypic characteristics. Two distinct subgroups make up G2.2.1: a subgroup from Spain (‘Mollar’ types) characterized by soft seeds with light pink arils and peel, and the CAC subgroup from Central Asia and California. The IL subgroup (G.2.2.2) has a common origin but is a phenotypically mixed group which splits into subgroups with strong phenotypic characteristics. Among these are the ERS subgroup characterized by red arils and peel, sweet taste and soft seeds, the “Black” subgroup (P.G.137–38 to P.G.127–28) with characteristic deep purple skin, and the “Hassas” subgroup characterized by pink arils and a weakly colored peel. The accessions in this latter subgroup were collected mostly from northern Israel and two accessions originated from Turkey (P.G.163–64 and P.G.209–220). This subgroup shares geographical origin as well as the aforementioned phenotypic characteristics.

## Conclusions

The significance of this study lies in creating an infrastructure for the elaboration of breeding strategies and for genetic mapping. It was aimed at establishing a global view of genetic interrelations in the pomegranate germplasm. As a result, it potentially orients the origins of, and suggests interrelations among pomegranate accessions all over the world as the accessions analyzed included some introduced from distant geographical locations. Although several studies on the genetic structure of pomegranate germplasm have already been performed [16], [73], they were based on a small set of genetic markers and accessions. Moreover, the broad information revealed on the pomegranate transcriptome in this study provides a source for further research into gene-function identification and metabolic pathways.

## Supporting Information

Table S1 **Functional annotation of pomegranate consensus sequences.**
(XLSX)

Table S2 **List of SSR motifs in pomegranate transcriptome.**
(XLSX)

Table S3 **Pomegranate accessions from the ARO germplasm collection.**
(XLSX)
